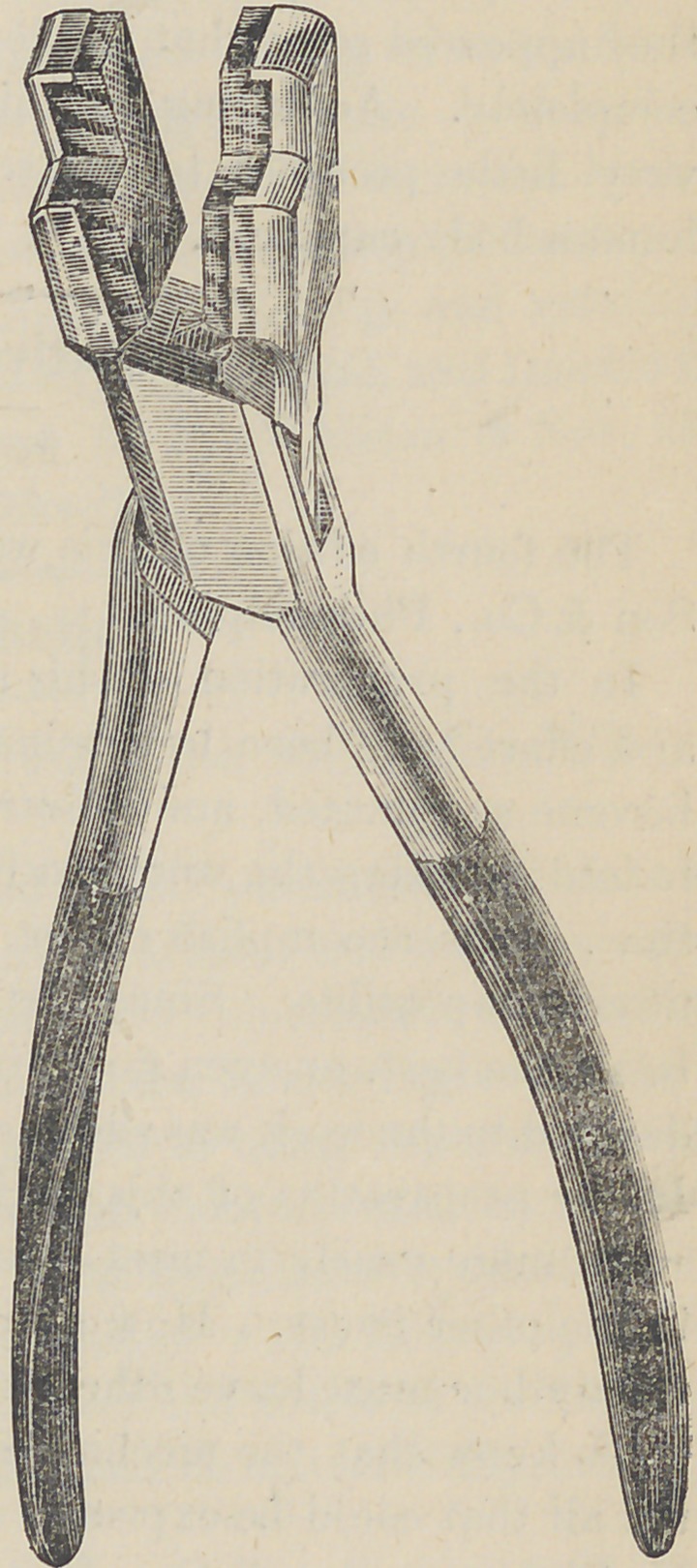# A Mechanical Forceps

**Published:** 1883-01

**Authors:** 


### A Mechanical Forceps.

The accompanying illustration shows a device of Dr. J. A. Throcmorton, of Sidney, O., for holding porcelain teeth while being ground for mounting on plates. The jaws of the instrument are lined with thick rubber which enables the teeth to be held firmly in any position, and that, too, without bending or injuring the pins. It is especially applicable for jointing blocks or single gum teeth, which can be done much more rapidly and accurately than by holding the teeth in the fingers, which is the usual method. The fatigue and grinding the fingers are also avoided. The instrument is a valuable one and should be in the laboratory of every dentist.

It can be obtained of Dr. Throcmorton, or at the dental depots.

## Figures and Tables

**Figure f1:**